# Internal Locus of Control and Sense of Coherence Decrease During the COVID-19 Pandemic: A Survey of Students and Professionals in Social Work

**DOI:** 10.3389/fsoc.2021.705809

**Published:** 2021-09-15

**Authors:** Melanie Misamer, Jörg Signerski-Krieger, Claudia Bartels, Michael Belz

**Affiliations:** ^1^ HAWK University of Applied Sciences and Arts, Göttingen, Germany; ^2^ Department of Psychiatry and Psychotherapy, University Medical Center, Göttingen, Germany

**Keywords:** mental health, locus of control, sense of coherence, feeling of powerlessness, coping strategies

## Abstract

Mental health is severely challenged by the COVID-19 pandemic due to a variety of restrictions in public and private life. Students in particular may face additional and unique stressors: face-to-face classes have been largely replaced by digital formats, leading to further reduced social contacts, thus facilitating the development of psychological symptoms. In this study, we aimed to assess the impact of the current peri-pandemic situation on students’ 1) locus of control and 2) sense of coherence, both of which have been linked to mental health in previous work. A total of 403 social work students from Germany participated, providing both retrospective (pre-pandemic) and current (February/March 2021) ratings. Furthermore, 324 social work professionals were included to analyze differences between both groups. Locus of control shifted significantly from internal to external during the pandemic for students and professionals. Furthermore, high mental burden correlated with increased external and decreased internal locus of control. Sense of coherence decreased during the pandemic for the entire sample and correlated with high mental burden. Overall, students showed a stronger drop compared to professionals, primarily due to a more pronounced decrease in perceived meaningfulness (all *p* < 0.001). In summary, students and professionals responded with increased feelings of powerlessness in the absence of sufficient coping strategies—this could lead to further deterioration of mental health as the pandemic continues. In this context, students appear to be particularly vulnerable to a reduction in sense of coherence. We conclude that interventions to improve coping strategies are urgently needed.

## Introduction

The COVID-19 pandemic has led to significant changes in public and private life, in part due to extensive restrictions during multiple lockdowns (Castiglioni and Gaj, 2020). These restrictions have severely challenged mental health in general (e.g., Kesner and Horáček, 2020). Because individual resources (in particular: social contacts) may subsequently be lost due to pandemic-related restrictions, individuals may become increasingly vulnerable to “spirals of loss” (Hobfoll, 2001), finally leading to an increased stress response. Accordingly, numerous studies point to a potential increase in mental disorders as a result of the pandemic, both for the general population (Wang et al., 2020; Rossi et al., 2020), and for health care workers (Tan et al., 2020; Kramer et al., 2021). The pandemic may also negatively impact general well-being (Jung et al., 2020), psychological resilience, and stress levels (McGinty et al., 2020).

The need for control is widely considered as a central human need (Grawe, 1998). Subjective loss of control during the pandemic, as a reaction to extensive restrictions and the unpredictability of the pandemic’s dynamics, has negative consequences on mental health, and also increases general stress levels and mental health problems (Kinman et al., 2020; Usher et al., 2020). In this regard, the psychological concept of *locus of control* represents a pertinent framework to operationalize the loss of control during the COVID-19 pandemic. It describes the extent to which individuals are convinced that they can control events themselves as a consequence of their own behavior (*internal* locus of control, in other words: gain of control), or feel powerless or controlled by external factors (*external* locus of control, in other words: loss of control) (Rotter, 1966). Over the past decades, a variety of studies have demonstrated a relationship between high internal locus of control and both mental and physical health (e.g., Jain and Singh, 2015; Kesavayuth et al., 2020). In the context of the COVID-19 pandemic, restrictive measures enacted by governmental authorities potentially shift feelings of locus of control from the internal to the external domain, and may result in negative consequences for mental health (Sigurvinsdottir et al., 2020; Alat et al., 2021).

Besides subjective loss of control, the individual ability to cope with the effects of the COVID-19 pandemic has been reported to have a major influence on mental health (Baloran, 2020). Routine coping mechanisms may prove useless during the pandemic and subsequently may lead to mental health problems (Barni et al., 2020). In this matter, the concept of *sense of coherence* (SOC) describes the individual ability to employ coping strategies to overcome a negative experience (Antonovsky, 1979). A prerequisite for successful coping is the perceived *manageability* of a situation, as well as its *meaningfulness* and the ability to understand the experience (*comprehensibility*). A pronounced sense of coherence has been associated with mental health and quality of life in numerous studies (e.g., Länsimies et al., 2017; Schäfer et al., 2018; del-Pino-Casado et al., 2019). Despite all efforts, individuals potentially perceive the pandemic and associated changes in daily life as being a great challenge to cope with. In this matter, pandemic-related restrictions may not always be perceived as being meaningful and/or completely understandable from the individual point of view. In this light, recent studies suggest that—apart from perceived locus of control—there is a negative relationship between sense of coherence and the COVID-19 pandemic. As a result, psychological stress levels may increase and mental health may deteriorate significantly (Gómez-Salgado et al., 2020; Schäfer et al., 2020; Leung et al., 2021; Ruiz-Frutos et al., 2021).

Young people and students in particular may face unique stressors during the course of the pandemic, as several empirical studies have shown. In general, face-to-face teaching has been widely replaced by digital teaching—this usually results in an additional reduction of social contacts and exchange opportunities for students (Chaturvedi et al., 2021). Most studies noted an increase of psychological symptoms in the context of the pandemic and frequently reported increased stress, anxiety, and/or depressive symptoms (e.g., Wang et al., 2020; Son et al., 2020; Meda et al., 2021). Elmer et al. (Elmer et al., 2020) suggested that COVID-19-specific concerns (including concerns about isolation, limited social networks, family health) may substantially contribute to these findings. Furthermore, there is evidence of increased anxiety about the future and lower well-being, particularly among undergraduate students (Dodd et al., 2021). In addition, online teaching potentially leads to a higher time burden for students and thus acts as an additional stressor (Dost et al., 2020).

Irrespective of the COVID-19 pandemic, it has also been shown for students that a pronounced internal locus of control is generally associated with better mental health (e.g., Sidola et al., 2015; Kurtović et al., 2018). However, studies on the specific influence of the COVID-19 pandemic on the perceived locus of control in students are not yet available. For sense of coherence, one previous study in nursing students has shown that it has been significantly less pronounced during the pandemic (Reverté‐Villarroya et al., 2021).

According to the empirical results so far, it is to be expected that students react to pandemic-related restrictions with pronounced feelings of powerlessness in the sense of 1) a reduced internal locus of control and 2) a reduced sense of coherence (and subsequently with mental burden). However, studies assessing the extent to which students are vulnerable in the context of the pandemic are scarce. The present cross-sectional survey aims to fill this research gap. The following hypotheses are empirically tested based on data from 403 social work students. The aim is to examine differences between retrospectively assessed (before the COVID-19 pandemic) and current experiences (approximately 1 year after the pandemic began, i.e., February/March 2021), with respect to locus of control and sense of coherence:

*H1a:* Students’ internal locus of control was higher before the COVID-19 pandemic than during the pandemic (February/March 2021).
*H1b:* Students’ external locus of control was lower before the COVID-19 pandemic than during the pandemic (February/March 2021).
*H2:* Students’ sense of coherence in students was higher before the COVID-19 pandemic than during the pandemic (February/March 2021).

Since there is only insufficient evidence on pandemic-related vulnerability among students in comparison to professionals to date, this study also surveyed 324 social work professionals for all primary endpoints to uncover possible differences between both groups.

## Methods

### Sample and Study Design

A Germany-wide cross-sectional online questionnaire study was conducted with social work students and professionals from February 17 to March 7, 2021, via the SoSci platform (SoSci Survey GmbH). The aim of the survey was to capture both the 1) *current* ratings on the selected scales on locus of control and sense of coherence during the COVID-19 pandemic (February/March 2021) and the 2) *retrospective* assessment of the participants before the COVID-19 pandemic (see *Measurement* for details). The use of retrospective measures is a well-established method (Suar et al., 2015). Especially during the COVID-19 pandemic which makes it difficult to conduct prospective studies it is a useful and feasible approach to assess pandemic-related changes (Bäuerle et al., 2020; Van Rheenen et al., 2020; Belz et al., 2021; Robillard et al., 2021). From March 8, 2021, the so-called nationwide “second lockdown” ended in Germany and was followed by easing of pandemic-related restrictions (Federal Government Germany, 2021). The survey was stopped from that date to avoid systematic bias.

Acquisition of online questionnaires was carried out nationwide via three routes: 1) social networks and platforms (e.g., Facebook, XING, or Telegram): Closed online groups and communities consisting of members from the field of social work were selected for acquisition (e.g., “Social Work,” “Critical Social Work”, and especially student groups from universities). 2) Universities in Germany: E-mail distribution lists for social work students at the University of Applied Sciences and Arts (HAWK) in Göttingen, Hildesheim and Apollon University in Bremen were used. 3) Social work institutions: Specific areas represented by social workers were contacted by e-mail (e.g., residential group areas of the AWO or youth welfare of the Diakonie).

In sum, 881 persons used the link to the online survey. Data entered analysis if the following criteria were fulfilled: 1) data completeness (primary endpoints), and 2) either studying or working in the field of social work. Such, *N* = 727 participants could be included in the study (82.5%). The study was approved on February 15, 2021 by the ethics committee of the University of Applied Sciences and Arts (HAWK) containing the a priori defined hypotheses (1a/1b/2). The survey was completely anonymous; thus, obtaining informed consent was not necessary.

### Measurement

In addition to the primary endpoints (see below), demographic information was recorded (age, gender, field of study/semester or occupation). Furthermore, the survey contained the self-developed, exploratory item “Do you currently feel mentally burdened?” to be answered on a scale from 1 = “not at all” to 5 = “very much”. This item was added to the questionnaire by request of the local ethics committee during the approval process and is not part of a validated scale. It was used as a control item to generally assess the amount of subjective mental burden of participants, and whether subjective burden correlates with our primary outcomes. It has been recently shown that mental burden is highly correlated with symptoms of a stress response (Belz et al., 2021).

#### Internal and External Locus of Control

The questionnaire *Internal-External-Locus of Control* (IE-4, Kovaleva et al., 2014) was used to measure the individually perceived locus of control. Here, two items each measure the scales internal and external locus of control (each subscale score is built by averaging the two scale items). For this purpose, the participants provide ratings of statements such as “I’m my own boss.” on a 5-point Likert scale (1 = “not at all true” to 5 = “completely true”) (see Table 1 for formulations of all items). In the context of the present study, the items of IE-4 were asked twice: once with reference to the “period before the pandemic” (retrospectively), and once with reference to “the current state” (February/March 2021). Apart from this introductory instruction, the items were not modified.

**TABLE 1:** Assignment of the translated questionnaire items to the hypotheses (primary endpoints).

**Hypotheses 1a/1b: Internal and external locus of control (IE-4)**	**Answer options**
Introductory text: “How do you experience your personal situation in general? […]”	
1a: “I’m my own boss.”	Likert scale^1^
1b: “If I work hard, I will succeed.”	
1c: “Whether at work or in my private life: What I do is mainly determined by others.”	
1d: “Fate often gets in the way of my plans.”	
**Hypothesis 2: Sense of coherence (Work-SoC)**	**Answer options**
Introductory text: “How do you experience your personal study/work situation? […]”	
2a^3^: “Unmanageable” vs “manageable”	Semantic differential^2^
2b: “Pointless” vs “meaningful”	
2c^3^: “Chaotic” vs “structured”	
2d^3^: “Uninfluenceable” vs “influenceable”	
2e: “Insignificant” vs “significant”	
2f^3^: “Unclear” vs “clear”	
2g^3^: “Uncontrollable” vs “controllable”	
2h: “Not worthwhile” vs “worthwhile”	
2i^3^: “Unpredictable” vs “predictable”	

*Notes.* All items were answered twice, for (a) the “period before the pandemic”, (b) the “current period” (February/March 2021). ^1^Likert scale (1 = “strongly disagree”; 5 = “strongly agree”), subscales: *internal locus of control* (1a/1b), *external locus of control* (1c/1d); ^2^Semantic differential (7-point scale with anchors listed in the table), ^3^inverted in the questionnaire, subscales: *manageability* (2d/2g), *meaningfulness* (2b/2e/2h), and *comprehensibility* (2a/2c/2f/2i).

#### Sense of Coherence

The *Work-Sense-of-Coherence questionnaire* (Work-SoC, Bauer et al., 2015) was used to assess sense of coherence. A total of nine items measure work-related sense of coherence (total score: average of all items). Participants give ratings of their work situation on 7-point semantic differentials, each with opposing anchor points (e.g., “manageable” vs “not manageable”; see Table 1 for all formulations). In addition to the total score, three subscales can be formed by averaging the corresponding items: manageability (2 items), meaningfulness (3 items), and comprehensibility (4 items). The items of the Work-SoC were also used twice in the present study, with reference to the “period before the pandemic” (retrospective) and “the current period” (February/March 2021). In addition, the introductory text asked about the “individual *study or work* situation” to specifically address students as well as professionals. Apart from that, the items remained unchanged.

### Statistical Analysis

SPSS^®^ statistical software (version 26) was used for data analysis. Descriptive representation of the variables was accomplished using means (*M*), mean differences (*M*_*Diff*_), standard deviations (*SD*), frequencies (*Freq.*), and Pearson correlations (*r*)^1^.

As primary endpoints of this study, differences between retrospective and current ratings were analyzed for a total of six scales. Hypothesis 1a/1b: 1) *internal* and 2) *external* locus of control (IE-4), and Hypothesis 2: sense of coherence (Work-SoC); 3*) total score* and three subscales: 4) *manageability*, 5) *meaningfulness*, and 6) *comprehensibility*. Six *t-*tests for dependent measures were used to analyze differences between retrospective and current ratings exclusively for the student subsample, along with corresponding effect sizes (Cohen’s d: *d*_*emp*_).

To analyze possible differences between students and professionals, a general linear model for repeated measures (GLM) was created for each of the six scales. The participants’ ratings on the scales (“retrospective” vs “current”) were integrated as a two-stage within-subject factor. In addition, the occupational situation (“student” vs “professional”) was integrated into each GLM as a two-stage between-subjects factor. Furthermore, within each GLM, the interaction effect between both factors was tested in order to map possible different trajectories between students and professionals on the individual scales. In order to statistically validate possible interaction effects, testing was conducted between both subgroups by means of two pairwise comparisons each at the retrospective and current time point (*t*-tests).

Besides the *t-*tests for the student-sample (6 tests), each GLM contained three *F*-tests and two pairwise comparisons (6 GLM × 5 tests = 30 tests). Because of α-error inflation, all *p*-values reported for the primary outcomes were globally adjusted using the Bonferroni method for the total number of 36 statistical tests. The initial significance level was set at *p* < 0.05 (two-tailed). For additional explorative analyses, the *p*-values were not corrected.

As the Work-SoC scale specifically asked for ratings on the “individual study or work situation”, only students who were at least in the third semester of a bachelor’s degree were included, since otherwise no retrospective statements could be made about their study situation before the COVID-19 pandemic. In this context, the available total *N* was reduced from 727 to 648 (see degrees of freedom of the statistical tests).

## Results

### Sample and Descriptive Results

See Table 2 for a summary of descriptive results. Of *N* = 727 participants, *n* = 613 (84.3%) were female and *n* = 110 (15.1%) were male. In addition, *n* = 4 (0.6%) reported gender as “diverse.” There were *n* = 403 (55.4%) students, and *n* = 324 (44.6%) professionals. The vast majority of students were in the bachelor’s program (*n* = 368, 91.3%; master’s: *n* = 35, 8.7%). The mean age of the total sample was *M* = 31.06 years (*SD* = 9.57). On average, students were 9.56 years younger (*M* = 26.81) than professionals (*M* = 36.37; *t* (724) = 15.38, *p* < 0.001). Mental burden resulted in ratings of *M* = 3.61 (*SD* = 0.96) for the total sample, thus tending to reach 4 = “quite burdened”. There was no statistically significant difference between students and professionals in terms of mental burden (*t* (722) = 1.58, *p* = 0.12, not significant: *ns*).

**TABLE 2:** Correlations and descriptive results.

***Variable***	**1**	**2**	**3**	**4**	**5**	**6**	**7**	**8**	**9**	***M* ± *SD*/*Freq***
**Sociodemographic variables**										
1. Student vs professional	–									s: 403. p: 324
2. Gender (binary)	−0.031	–								m: 110. f: 613
3. Age	−0.496^**^	−0.065	–							31.06 ± 9.57
4. Current mental burden	0.059	0.087^*^	−0.052	–						3.61 ± 0.96
**Locus of control** ^**1**^										
5. Δ^1^ Internal locus of control	−0.020	−0.057	0.035	−0.426^**^	–					−0.72 ± 0.82
6. Δ^1^ External locus of control	0.039	0.059	−0.099^**^	0.299^**^	−0.493^**^	–				0.59 ± 0.88
**Sense of coherence** ^**2**^										
7. Δ^2^ Manageability	−0.061	−0.062	0.017	−0.239^**^	0.375^**^	−0.301^**^	–			−1.69 ± 1.66
8. Δ^2^ Meaningfulness	−0.240^**^	0.027	0.184^**^	−0.276^**^	0.366^**^	−0.209^**^	0.364^**^	–		−1.03 ± 1.50
9. Δ^2^ Comprehensibility	−0.151^**^	−0.065	0.090^*^	−0.402^**^	0.447^**^	−0.318^**^	0.605^**^	0.429^**^	–	−1.69 ± 1.43
10. Δ^2^ Total score	−0.196^**^	−0.042	0.128^**^	−0.396^**^	0.498^**^	−0.343^**^	0.769^**^	0.745^**^	0.882^**^	−1.47 ± 1.21

*Notes.* Correlations: *p < 0.05; **p < 0.01; *M* = mean; *SD* = standard deviation; *Freq.* = frequency; student = 1 vs professional = 0; gender (male = 1, female = 2); mental burden = values from one to 5 (“not at all” to “very much”); *Δ* = delta (*Δ* = (current status)—(pre-pandemic status)) of questionnaire scales: ^1^IE-4 (values from 1 to 5, *N* = 727); ^2^Work-SoC (value from 1 to 7, *N* = 648).

Correlations between the variables are shown in Table 2. The status “student” compared to “professional” correlated with a more pronounced decrease in sense of coherence (Work-SoC) from the pre-pandemic to the current state: Significance was achieved for the Work-SoC total score as well as the subscales meaningfulness and comprehensibility (*r* between −0.151 and −0.240, all *p* < 0.001). High mental burden also correlated with negative changes in the sense of coherence for all (sub-) scales (*r* between −0.239 and −0.402, all *p* < 0.001), as well as with decreasing internal locus of control (*r* = -0.426, *p* < 0.001) and increasing external locus of control (*r* = 0.299, *p* < 0.001).

### Locus of Control (Hypothesis 1)

**FIGURE 1:**
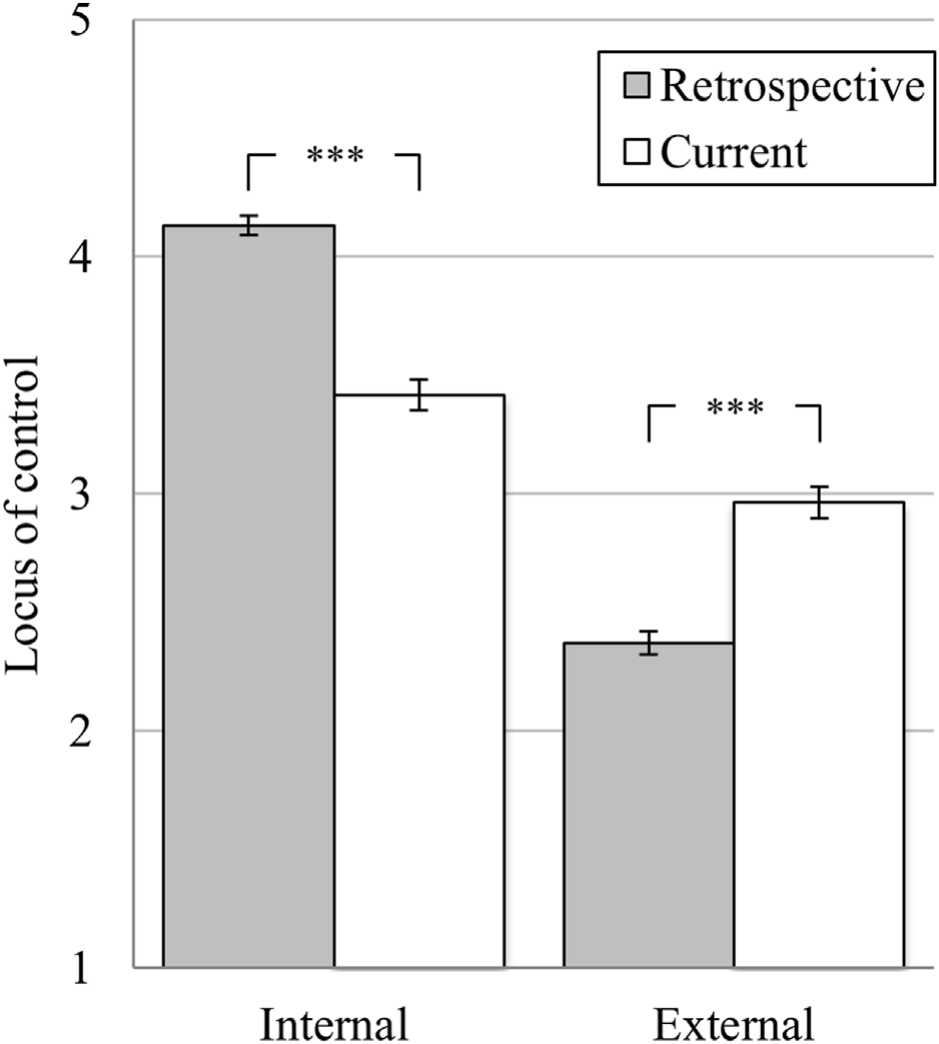
Change in internal and external locus of control. *Notes:* *p < 0.05; **p < 0.01; ***p < 0.001; means with 95% confidence intervals (1 = “strongly disagree” 5 = “strongly agree”); *retrospective* = retrospective rating related to a pre-pandemic time-point; *current* = assessment at the current time point (February/March 2021). *N* = 727.

Students rated their internal locus of control significantly higher at the pre-pandemic (*M* = 4.15, *SD* = 0.56) than at the current time point (*M* = 3.42, *SD* = 0.88; *t* (402) = 17.10, *p* < 0.001, *d*_*emp*_ = 0.85). Thus, the assumption made in Hypothesis 1a, that internal locus of control would decrease in students during the COVID-19 pandemic, is accepted. Also, internal locus of control was rated significantly higher at the pre-pandemic (*M* = 4.13, *SD* = 0.55) than at the current time point (*M* = 3.42, *SD* = 0.88, GLM: *F* (1, 725) = 541.13, *p* < 0.001, partial η^2^ = 0.43 see Figure 1) by the entire sample. Neither a general difference between professionals and students could be found (between-group effect: *F* (1, 725) = 0.37, *ns*) nor different trajectories between the two groups (interaction effect: *F* (1, 725) = 0.29, *ns*, all pairwise comparisons *ns*).

External locus of control was rated significantly lower by students at the pre-pandemic (*M* = 2.42, *SD* = 0.69) compared to the current time point (*M* = 3.04, *SD* = 0.91; *t* (402) = 13.92, *p* < 0.001, *d*_*emp*_ = 0.69). The assumption that external locus of control would increase among students during the COVID-19 pandemic (Hypothesis 1b) is accepted. External locus of control was also rated lower at the pre-pandemic (*M* = 2.37, *SD* = 0.69) compared to the current time point (*M* = 2.96, *SD* = 0.92, GLM: *F* (1, 725) = 320.05, *p* < 0.001, partial η^2^ = 0.31 see Figure 1) by the entire sample. Again, neither a different course between students and professionals (interaction effect: *F* (1, 725) = 1.09, *ns*, all pairwise comparisons *ns*), nor a general difference between both groups (between-group effect: *F* (1, 725) = 7.61, *ns*) could be found.

### Sense of Coherence (Hypothesis 2)

Students rated the sense of coherence (Work-SoC: total score) significantly higher at the pre-pandemic (*M* = 5.51, *SD* = 0.72) than at the current time point (*M* = 3.80, *SD* = 1.09; *t* (232) = 23.13, *p* < 0.001, *d*_*emp*_ = 1.28). Comparable reductions were also found for all three subscales (manageability: *M*_*Diff*_ = −1.71, meaningfulness: *M*_*Diff*_ = −1.39, comprehensibility *M*_*Diff*_ = −1.90; all *p*-values < 0.001). Thus, the assumption made in Hypothesis two that the sense of coherence would decrease in the COVID-19 pandemic for students is accepted for the total score and additionally for all three subscales.

**FIGURE 2:**
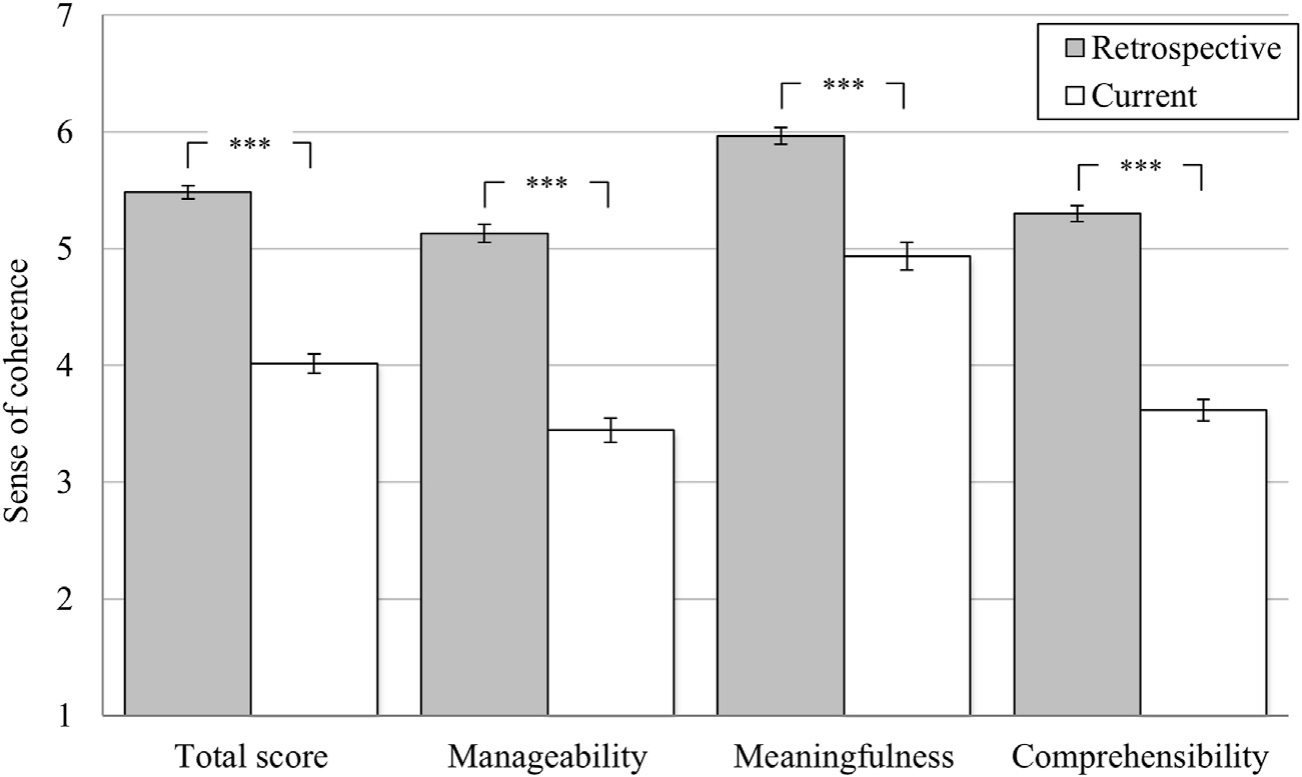
Change in sense of coherence including subscales. *Notes:* *p < 0.05; **p < 0.01; ***p < 0.001; means with 95% confidence intervals (7-point semantic differentials; see Table 1 for item anchors); *retrospective* = retrospective rating related to a pre-pandemic time-point; *current* = assessment at the current time-point (February/March 2021). *N* = 648.

**FIGURE 3:**
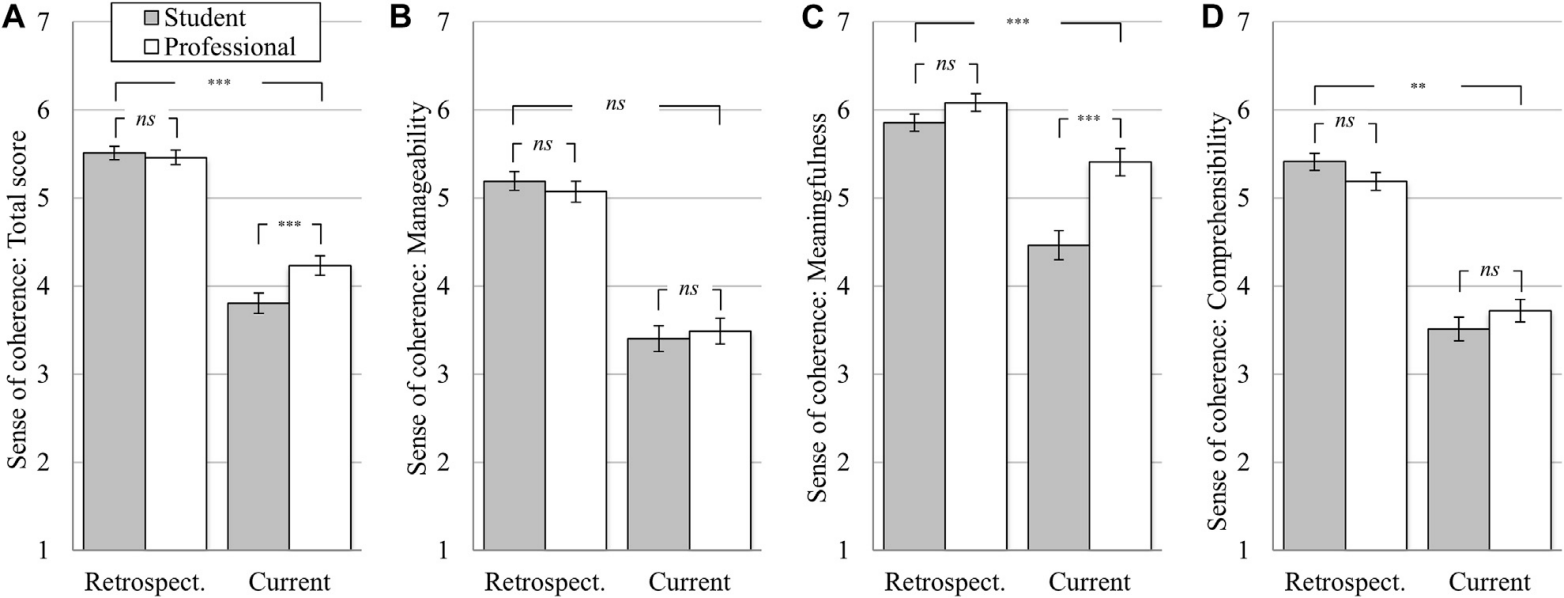
Change in sense of coherence differentiated between students and professionals; **(A)**: total score, **(Β)**: manageability, **(C)**: meaningfulness, **(D)**: comprehensibility. *Notes*: between groups (students vs professionals) and interaction effects; *p < 0.05; **p < 0.01; ***p < 0.001; means with 95% confidence intervals (7-point semantic differentials; see Table 1 for item anchors); *retrospective* = retrospective rating related to a pre-pandemic time-point; *current* = rating of the current time-point (February/March 2021); *students* (*n* = 324) vs *professionals* (*n* = 324); *N* = 648.

Also, the entire sample rated sense of coherence (Work-SoC: total score) significantly higher at the pre-pandemic (*M* = 5.48, *SD* = 0.74) than at the current time point (*M* = 4.02, *SD* = 1.07, GLM: *F* (1, 646) = 978.67, *p* < 0.001, partial η^2^ = 0.60 see Figure 2). Again, reductions from the retrospectively assessed time point compared to the current time point were also found for all three subscales of the Work-SoC questionnaire (manageability: *M*_*Diff*_ = −1.69, meaningfulness: *M*_*Diff*_ = −1.03, comprehensibility *M*_*Diff*_ = −1.69). All reductions in the subscales reached significance (GLM: *F* (1, 646) from 326.37 to 918.61, all *p*-values < 0.001, see Figure 2). A significant interaction effect was found for the total score (GLM: *F* (1, 646) = 25.71, *p* < 0.001, partial η^2^ = 0.04 see Figure 3A). While students and professionals did not differ at the retrospectively assessed pre-pandemic time point (*M*_*Diff*_ = 0.05, *t* (646) = 0.88, *ns*), students reported a significantly lower sense of coherence than professionals at the current time point (*M*_*Diff*_ = 0.42, *t* (646) = 5.16, *p* < 0.001). For the subscales manageability and comprehensibility, this interaction effect could either not be found, or could not be corroborated by significant pairwise comparisons (see Figure 3B/D). For the subscale meaningfulness, a significant interaction effect was found (GLM: *F* (1, 646) = 39.44, *p* < 0.001, partial η^2^ = 0.06 see Figure 3C). Again, students and professionals did not differ in their retrospective, pre-pandemic assessment (*M*_*Diff*_ = 0.23, t (646) = 3.14, *ns*), but students rated the experienced meaningfulness significantly lower than professionals at the current, peri-lockdown time point (*M*_*Diff*_ = 0.94, *t* (646) = 8.18, *p* < 0.001).

## Discussion

The present cross-sectional study investigated whether the COVID-19 pandemic has a negative influence on perceived internal locus of control as well as on the sense of coherence in social work students. For this purpose, retrospective ratings about the time before the pandemic were compared with ratings approximately 1 year after the beginning of the pandemic (February/March 2021). To analyze whether these students are specifically vulnerable to experience these changes, we compared ratings of 403 students to those of 324 professionals.

### Central Findings of This Study

As postulated in hypotheses 1a/1b, the internal locus of control decreased significantly in the population of students during the COVID-19 pandemic, while the external locus of control increased in parallel. No difference between students and professionals could be found; both showed a similar, strong response in the context of the pandemic. Furthermore, an increased mental burden was strongly correlated with a shift from internal to external locus of control. In accordance with previous work, participants interviewed here might have reacted with a strong increase of mental burden due to the experience of powerlessness with regard to their own life organization during the pandemic (Jain and Singh, 2015; Kesavayuth et al., 2020; Sigurvinsdottir et al., 2020; Alat et al., 2021). In other words, their need for control as a central human need (Grawe, 1998) may have been neglected due to pandemic-related restrictions. Hence, a persistently high level of subjective powerlessness could intensify mental health problems and general stress levels in the future (Kinman et al., 2020; Usher et al., 2020). Our results also support pre-pandemic findings, suggesting a relationship between internal locus of control and mental health (Jain and Singh, 2015; Kesavayuth et al., 2020): Accordingly in this study, internal locus of control was negatively correlated—in contrast to external locus of control—with mental burden.

As postulated in hypothesis 2, the sense of coherence decreased among students during the pandemic. Similarly, a significant drop was found for professionals. One interpretation would be that neither students nor professionals have sufficient coping strategies to deal with pandemic-related challenges and are thus unable to cope adequately with this stressor (Antonovsky, 1979). This may also be accompanied by serious consequences for mental health (Länsimies et al., 2017; Schäfer et al., 2018; Baloran, 2020; Barni et al., 2020; Leung et al., 2021). For sense of coherence, it has to be considered that this concept was originally defined as a developmental construct or a dispositional orientation (Antonovsky, 1979; Hammond and Niedermann, 2010), and would therefore imply to remain essentially unchanged by environmental factors or interventions. In contrast, several empirical studies showed that sense of coherence within a person is not a stable trait per se. Thus, it has been shown to undergo changes (Smith et al., 2003; Feldt et al., 2011), and does not reach stability depending on a certain age (Feldt et al., 2003). With results of a significantly decreasing sense of coherence during the COVID-19 pandemic, the original assumption is challenged. Thus, the present study supports the idea that environmental conditions can change the individual sense of coherence.

It is of particular note that among students both the total score for sense of coherence and, more specifically, the sense of meaningfulness dropped more sharply than among professionals during the pandemic. These findings may be interpreted as specific vulnerability and bear important implications: A connection between subjective meaningfulness and individual work engagement during the COVID-19 pandemic has already been shown for healthcare workers (Liu et al., 2021). In their study, the implementation of a combined organizational intervention (a supportive letter that stressed the subjective meaningfulness and crisis management via counseling sessions) led to increased individual work engagement. One may thus consider that students who perceive their study to be more meaningful and the COVID-19 pandemic as less interfering would also profit from such interventions and increase their engagement. Consequently, subjective study-related meaningfulness should be increased via the promotion of sense-making processes, and may also lead to a reduction of mental burden in students. Fostering of meaning-making processes (“normalization”) in light of the pandemic (i.e., recognition that stress reactions are normal given the current situation) represents a promising approach for psychological intervention (Castiglioni and Gaj, 2020): Promoting the understanding of stressful experiences can be considered as an important coping strategy and can substantially contribute to improving sense of coherence. Complementary pandemic-compatible strategies to improve mental health are already available, such as sports psychology interventions (Bertollo et al., 2021), specific pandemic-related training and preparation (Reverté-Villarroya et al., 2021), and the use of interventions such as mindfulness and a reduction of news consumption (Aughterson et al., 2021).

### Limitations

First, in the present cross-sectional study, the assessment of locus of control and sense of coherence was conducted both retrospectively and at a time point approximately 1 year after the beginning of the pandemic, but ultimately it was a post hoc survey. Retrospective assessment is generally more subject to measurement error because it is retrieved from memory and additionally influenced by each participant’s personality traits (Ottenstein and Lischetzke, 2020). Accordingly, data must be interpreted with caution. Recently, Belz et al. have shown that a retrospective, questionnaire-based measurement during the COVID-19 pandemic can yield good objectivity, reliability and validity (Belz et al., 2021). In sum, a longitudinal design would minimize this measurement error but was not feasible due to the unpredictability of the pandemic development. Second, the questionnaire was deliberately designed to be short so as not to deter participants from the outset—the mean completion time (min) was *M* = 4.84 (*SD* = 1.91) with a completion rate of 82.5%. Based on the large sample size acquired, it would have been statistically reasonable to collect more items/scales to gain more insights—however, this would have led to a potentially higher dropout rate at the same time. In this light, we only used a single, self-developed item to assess mental burden of participants (“Do you currently feel mentally burdened?”; 1 = “not at all” to 5 = “very much”). This can be criticized, as—in contrast to the primary outcomes of this study—this item was not derived from an established scale, but simply formulated to record a self-report of mental burden post hoc. The item is thus not theory-driven, which is a clear limitation of this study and why it was only included in the exploratory analysis. In the future, established scales covering multiple facets should be used, even at the cost of a slightly longer questionnaire. Third, although the representativeness of the sample studied here can be considered potentially high due to the large sample size, the survey was conducted online exclusively. This may subject the dataset to inclusion biases that cannot be controlled. It should be noted at this point that due to the increased digitization during the pandemic (e.g., digital teaching, home office) more people have potentially acquired competencies in this area and can thus be widely reached by an online survey. This would in turn be indicative of a good sample representativeness. Fourth, we chose scales to assess locus of control and sense of coherence in this study. Undoubtedly, there are numerous resources which can help to cope with critical life events—like personal traits (e.g., self-efficacy) or energy resources (e.g., money; please see (Hobfoll and Buchwald, 2004) for details). These were neither empirically investigated nor statistically controlled here. It would be worthwhile to consider the relationship between such resources and the main outcomes focused here in future empirical studies.

## Conclusion

Social work students and professionals react to the COVID-19 pandemic with increased feelings of powerlessness. Due to the currently unpredictable continuation of the pandemic, the negative effects found here may further escalate. In view of an increased vulnerability of students for a deteriorated sense of coherence, longitudinal observations of mental health are urgently needed for this population that has been insufficiently considered so far. Accordingly, the need for appropriate and population-specific intervention strategies would be uncovered.

## Data Availability

The raw data supporting the conclusions of this article will be made available by the authors, without undue reservation.

## Footnotes

1 The four-field correlation coefficient (phi coefficient, r) was calculated for the correlation of the binary variables “student” vs “professional” and “binary gender” (“male” vs “female”).
